# Local Characteristics Shape the Intended Political Behaviours of Adolescents

**DOI:** 10.1007/s11205-021-02852-y

**Published:** 2021-12-06

**Authors:** Daniel Deimel, Hermann J. Abs

**Affiliations:** Faculty of Educational Sciences, University of Duisburg-Essen, 45141 Essen, Germany

**Keywords:** Regional characteristics, Political socialisation, Intended political behaviour, Conventional political participation, Political protest

## Abstract

Among the different factors that predict political participation, the characteristics of the local community are often described. The types and the intensity of political participation differ in urban and rural communities. The local community provides social and cultural resources for political participation and at the same time acts as a driver of political socialisation. The political attitudes of adolescents differ depending on the characteristics of the communities in which they live, i.e. the local context of their political socialisation. This paper describes the context of the political socialisation of adolescents in the German federal state of North Rhine-Westphalia (NRW) in terms of geographical differences in socioeconomic, sociostructural, and sociocultural characteristics. The approach uses public databases to construct indicators that describe administrative districts in terms of their degree of urbanisation, degree of aggregated individual wealth, and variety of opportunity. These indicators were merged with the NRW subset of the International Civic and Citizenship Education Study (ICCS 2016), which comprises *N* = 1451 students in *N* = 59 schools. Neither the degree of urbanisation nor the degree of aggregated individual wealth was suitable for explaining differences in the intended political behaviour of secondary school students in the 8th grade in NRW. However, the higher the variety of opportunity in a certain district, the less frequently students intend to participate in elections as adults. Also, the higher the measure, the more frequently students intend to participate in illegal protest activities. Apparently certain local environments mobilise intentions to participate in protest activities and suppress more conventional political activities.

## Introduction

Democratic societies legitimise themselves by providing opportunities for political participation (Dahl, 1989). In its broadest definition, political participation is described as a continuously expanding repertoire of activities aiming to defend interests, express opinions in public, and influence the decisions of authorities (Theocharis & van Deth, 2018). However, intentions to participate and other political orientations differ between age cohorts. In order to preserve the democratic process for the future, a better understanding of the political socialisation of adolescents is needed (Hooghe, 2004).

Among the different factors that predict political participation, the characteristics of the local community are often considered. These characteristics refer to socioeconomic, sociostructural, and sociocultural differences between local communities and provide a local context for political behaviours: how do individuals participate politically and to what extent? For example, voter turnout, party preferences, and participation in political organisations differ in urban and rural communities (Holtmann, 2019; Milbrath, 1972; Monroe, 1977; Rodden, 2019; Verba & Nie, 1972). Unconventional political activities such as protesting are more prevalent in bigger cities (Hasson, 1997; Schoene, 2018). In general, urban environments have been described as obstacles to the development of civic competences and political behaviours (Hart & Atkins, 2002). Such observations are related to the underlying social structure of these communities. As political behaviours are said to crystalise during adolescence and remain relatively stable over an individual’s lifetime (Neundorf & Smets, 2017), it is crucial to identify these structural factors as drivers for political socialisation. Disparities between local communities can predict the levels of adolescents’ performance in school (Jeworutzki & Schräpler, 2019). However, only little is known about how these differences shape the political orientations of adolescents, and the respective results are inconclusive (Coopmans et al., 2020; Schulz et al., 2010, 2018a, 2018b).

To address this research gap, this paper pursues two aims: firstly, to develop indicators that adequately depict the local context of adolescents’ political socialisation; and secondly, to describe how these indicators relate to outcomes of political socialisation, especially adolescents’ intentions regarding their political participation. The development of these indicators is based on a literature review which explores the local contexts of adults’ political participation and adolescents’ political socialisation. We will study the case of the German federal state of NRW using a combination of data taken from open databases provided by governmental entities (IT.NRW, 2020a, b) and the 2016 International Civic and Citizenship Education Study (ICCS 2016; Schulz et al., 2018b), an international large-scale assessment that analyses the outcomes and contexts of the political socialisation of adolescents.

## Background

To obtain a firm understanding of the local context of political socialisation, we will review the current state of research on patterns of political participation emerging in different local environments. In particular, we will seek to explore in what ways and for what reasons political participation differs between citizens who reside in cities, suburbs, and rural communities. Building on the existing research on the socioeconomic, sociostructural, and sociocultural differences between communities as contextual determinants of political participation, we will infer how these reasons are linked to differing local conditions for the political socialisation of adolescents.

### The Local Context of Adults’ Political Participation

Discussions on the local context of adults’ political participation have traditionally focused largely on the differences in opportunities and networks provided in cities versus smaller rural communities along two opposing axes—the *mobilisation model* and the *decline-of-community model*—but without necessarily considering socioeconomic factors. Milbrath (1972) formulated an environmental approach to explain the type and extent of political participation of citizens in different communities. In this view, people closer to a society’s centre are more likely to be involved in political participation than people closer to the periphery. They ‘receive more stimuli enticing them to participate, and they receive more support from their peers when they do participate’ (p. 114). Today, in times of digital citizenship, it is a matter of debate how a society’s centre can be defined (Choi et al., 2017), but for Milbrath writing in 1972, the urban–rural dimension was equivalent to the centre-periphery dimension. This model sees the size of a city predominantly as a proxy for the opportunities that it has to offer in terms of politicising stimuli, which is also described as the *mobilisation model* of politicisation (Verba & Nie, 1972). In contrast, however, the *decline-of-community model* posits that in larger entities, participation is more difficult because of greater complexity. Compared with smaller communities, ‘politics is more complicated, impersonal and distant’ (Verba & Nie, 1972, p. 231). Levels of participation decline in these larger communities, which also offer less social cohesion. Community involvement is less likely to be observed among residents of urban environments (Putnam, 2000). Therefore, networks that might engage citizens in political action are less likely to be accessed in larger cities. This might be a self-sustaining process: over several decades, a steady decline in organisational memberships has been observed (Putnam, 2000). Furthermore, this decline is closely related to suburbanisation; for example, commuting reduces the time citizens have available to become involved in their communities. At the same time, suburbanisation is related to spatial fragmentation between workplaces and homes. Social circles compete with each other, and networks that foster involvement are less likely to be sustained. ‘In short, sprawl is a collective bad, both for commuters and for stay-at-homes’ (Putnam, 2000, p. 214). In addition, Monroe (1977, p. 77) speculates that the decline of political engagement in more urban environments is related to the opportunities these environments offer: ‘[t]he modern urbanite […] has a myriad of competing attractions for his time and interest’. In this understanding, political participation is only one activity that individuals can pursue to fulfil their needs, and it seems to be easier to avoid involvement in communities in larger cities.

The above notwithstanding, the size of a city does not always seem to be an adequate predictor of political participation and should be examined independently from the socioeconomic status of its inhabitants (Verba & Nie, 1972). Considering the socioeconomic context shows that individual voting behaviour is likely to be determined by the social structure of the community in which individuals reside, which in turn is explained by self-selection (Harrop et al., 1991). The fact that two-career families are less involved in their communities (Putnam, 2000) might be related to this phenomenon as more traditional lifestyles might be more prevalent in non-urban environments (van Diepen & Musterd, 2009). At a contextual level, socioeconomic factors such as aggregated higher incomes and wealth are positively correlated with higher levels of voter turnout (Cancela & Geys, 2016; Nadeau et al., 2019). In turn, non-voting seems to be more prevalent in constituencies with less privileged sociostructural features (Holtmann, 2019). Further, past voter turnouts were found to be relatively strong predictors of future turnouts (Cancela & Geys, 2016). In short, areas that host citizens with higher incomes and that showed higher voter turnouts in the past are more prone to fostering future political participation.

Additionally, not all forms of participation are affected by the decline of communities, and more disadvantageous social structures do not necessarily lead to a decline in participation. While organisational memberships and community activities that rely on informal cooperation between citizens suffer from suburbanisation and the lack of cohesion in larger cities, protest activity does seem to emerge in these (Hasson, 1997; Schoene, 2018). Taking a critical perspective, Nicholls (2008) describes cities as important locations for protest activities because marginalised groups have fewer means of exercising conventional political power. For marginalised groups, organisational involvement can foster political engagement (Verba et al., 1995). Cities might provide a larger base of networks particularly for marginalised groups. The features of urban communities therefore might mobilise individuals for some activities, such as protest, and at the same time lead to the decline of other, more conventional means of political participation (e.g. Barnes et al., 1979), such as voting.

In sum, the environment in which individuals reside impacts their societal and political participation. While adults’ opportunities for participation differ between urban, suburban, and rural communities, the relationship is not straightforward. This observation might extend to adolescents’ attitudes towards political participation because the communities in which they grow up offer different resources for their political socialisation.

### The Local Context of Adolescents’ Political Socialisation

The concept of the political socialisation of adolescents ‘describes the process by which citizens crystalize political identities, values and behavior that remain relatively persistent throughout later life’ (Neundorf & Smets, 2017, p. 1). Among other factors, families are focal agents especially of early political socialisation. ‘Socializing agents either directly or indirectly teach children about politics but also have a mobilizing function as they influence, encourage, or discourage young people’s political preferences and political action’ (p. 6). Direct and indirect influences have been assumed to impact the transmission of political orientations within families. Jennings et al. (2009) describe a direct transmission of parental political orientations due to the observation of political behaviours, discussions about political issues, and the exchange of political information within families. In highly politicised families, children are more likely to adopt their parents’ views because they experience consistent opportunities to shape their political orientations over a sustained period of time. A family’s socioeconomic status (SES) may well be directly related to this phenomenon (Hoskins & Janmaat, 2019). SES denotes the relative position of an individual within a society’s social structure. Children’s SES is determined by the occupational status and educational attainment of their parents (Sirin, 2005). Individual political knowledge and civic engagement are equally related to individual SES (Delli Carpini & Keeter, 1996), which means that social learning opportunities within families may differ according to their SES. Thus, the higher a family’s SES, the more frequent and richer the possible opportunities for political learning.

Intergenerational transmission further extends to structural factors such as social class, race, or religion. The location of children within the social structure is largely determined by their families. Children may face similar attitude-shaping experiences; therefore, their social position indirectly influences their political orientations (Glass et al., 1986). Chmielewski (2014) shows that students’ SES affects whether they enter academic or vocational tracks in education systems that make a formal distinction between those tracks. Students are further segregated by implicit tracking (Salchegger, 2016), whereby geographical location determines a school’s catchment area which, in turn, may affect which school track a child can access.

Students will mostly have peers with similar social backgrounds. This is especially the case for disadvantaged students in cities, where neighbourhood poverty was observed to be reproduced over time (van Ham et al., 2016). Janmaat et al. (2014) claim that the social and ethnic segregation of students may affect their future voting behaviour; students within disadvantaged contexts are less likely to participate politically. Direct and indirect influences on political socialisation in the family interact: students will most likely partake in these social learning occasions with peers who have faced similar political socialisation at home. Deimel et al. (2020) therefore hypothesised that access to resources of political socialisation is partly determined by geographical location. In educational research, there are several approaches to assess the availability of resources that influence the quality of socialisation processes. For example, the socioeconomic structure of the classroom (e.g. Hattie, 2002) or the immediate neighbourhood of schools (Jeworutzki & Schräpler, 2019; Schräpler & Jeworutzki, 2016) could impact educational outcomes. However, these approaches do not take into consideration explanations of differences that lie beyond the social composition of a group.

The local context provides social and cultural resources and may act as a driver of political socialisation. The political attitudes of adolescents should therefore differ depending on the characteristics of the communities in which they live. This effect should be especially visible in their political participation. However, the actual political behaviour of adolescents cannot be assessed adequately as adolescents are subject to legal restrictions (e.g. voting age). Instead, studies related to political socialisation therefore often observe behavioural intent. Adolescents’ intentions for political participation predict actual political participation (Eckstein et al., 2013; Glasford, 2008). In general, behavioural intentions as prerequisites of actual behaviours are partly determined by perceived social norms (Ajzen, 1991). Social norms differ by geographical location, which also fosters geographically differentiated modes and intensity of political participation (Putnam et al., 1993). Therefore, if social norms differ geographically and if they also predict behavioural intentions, adolescents’ intentions regarding their political participation should differ geographically. Depending on their geographical location, adolescents have different access to resources for political learning, experience different opportunities for shaping their political orientations, and internalise different social norms regarding political participation.

For this reason, the influence of the local community is included in the theoretical frameworks of studies that aim to explain the political socialisation of adolescents. For example, ‘schools and homes of students are located in communities that vary in their economic, cultural, and social resources, and in their organisational features. Inclusive communities […] may offer civic and citizenship opportunities for partnerships and involvement to schools and individuals’ (Schulz et al., 2016, p. 44). There is some evidence that adolescents’ civic skills are more prevalent in more urban environments (Coopmans et al., 2020). Schulz et al. (2010) also observed higher political knowledge among adolescents in urban environments at least in some countries or educational systems in the ICCS 2009 study; however, these results were not replicated in the 2016 study cycle (Schulz, et al., 2018a, 2018b). Furthermore, students in urban communities were described in the ICCS 2016 study as more tolerant and more averse to corruption when compared to students in rural communities in Latin America. The hypothesis that this pattern was related to sociostructural differences between urban and rural communities was not, however, empirically evidenced (Schulz, et al., 2018a, 2018b). It remains unclear from this research to what extent the local context drives adolescents’ political socialisation.

Looking back at the preceding two sections, we can identify two deficiencies in existing research. Neither does the size of a city alone adequately explain the variation in political behaviours of adults, nor does the local context explain differences in adolescents’ political socialisation. The empirical evidence does not fully support its theoretical foundation, especially regarding the intended political participation of adolescents. This paper seeks to address this discrepancy by attempting to narrow the gap between theory and evidence.

## Research Questions

As discussed above, the emergence of intended political behaviour can *in theory* be explained by its local context. This equally extends to sociostructural, socioeconomic, and sociocultural aspects. The local community should provide resources, networks, or structured opportunities for political participation. Although research on the political socialisation of adolescents theorises that such an influence does exist, it does not provide empirical evidence. This lack of evidence could result from the fact that the statistical models used in existing research focus on the degree of urbanisation without including other factors. We posit, however, that the characteristics of the communities in which adolescents grow up and their experiences of political socialisation act as local context that has to be taken into account when explaining the differences in their intentions to participate politically. In light of the preceding arguments, we pursue three research questions:

1. How can the local context of adolescents’ political socialisation be depicted adequately?
2. Is the degree of urbanisation the most influential driver of political socialisation when it comes to intentions regarding political participation?
3. To what extent do other sociogeographic differences explain differences in the intended political participation of adolescents?

## Methods

In this section, we will introduce the regional context of our study as well as the data sets and the methodological approaches used to identify indicators of the local context of political socialisation and the relationships between the indicators and the outcomes of political socialisation.

### Regional Context: North Rhine-Westphalia

In Germany, school education is the exclusive policy domain of the country’s sixteen constituent federal states. The national ministry of education has no legal influence on schooling in the federal states. Because the federal states differ in many aspects, we selected for our analysis the federal state which best reflects Germany as a whole, namely NRW. NRW provides an interesting regional context for our study for four reasons, as follows.

First, population size—NRW is the most populous of Germany’s sixteen federated states. Apart from the city-states of Hamburg, Bremen, and Berlin, it is the most densely populated area in Germany with over 500 people per square kilometre, totalling nearly 18 million citizens.

Second, rural–urban composition—The state consists of urban areas, like the city of Cologne or the Ruhr area, as well as rural areas such as the Eifel or Ostwestfalen.

Third, socioeconomic factors—The former coal and steel region of NRW still accounts for roughly one fifth of Germany’s total gross domestic product (GDP). While exceeding other federal states in size and economic power, NRW seems to represent the ‘average’ Germany very well. For example, the GDP per capita, the unemployment quota, or the share of non-German residents in NRW corresponds to the respective averages in the Federal Republic of Germany (DESTATIS, 2020).

Fourth, the school system—NRW has a tracked school system in which the allocation of students to one of the different school types occurs in the first year of secondary school education, i.e. in grade five, when children are typically around 11 years old (European Commission/EACEA/Eurydice, 2018). The highest and at the same time most popular type of secondary school in NRW is the academic track, which is attended by 40 per cent of all students in secondary education (IT.NRW, 2020b). Large disparities have been observed regarding the social composition of schools between rural and urban communities in NRW: schools in urban communities are more likely to deal with problems arising from social disparities (Schräpler & Jeworutzki, 2021). Further, the tracked school system in NRW is related to the social segregation of students, with visible differences in their political orientations (Deimel et al., 2020).

### Data Set 1: International Civic and Citizenship Education Study (ICCS 2016)

To depict the outcomes of adolescents’ political socialisation, we used a subsample of the 2016 International Civic and Citizenship Education Study (ICCS 2016, Schulz et al., 2018b). This international large-scale assessment asks how well 14-year-old students are prepared to undertake their role as active citizens in 24 regional entities worldwide. In most cases, countries participate in ICCS as a whole, like Denmark or Italy. In other cases, smaller regional entities participate if they have autonomous legislative power in the domain of education for their region (like Flanders in Belgium or NRW in Germany). In Germany, the ICCS 2016 study included only the federal state of NRW. In NRW, a multistage randomised sample with 167 schools was drawn. Participation was decided at school level. For each school, up to two replacement schools were selected in case the originally sampled or the first replacement school declined participation in the study. This resulted in a sample of 1451 students from 59 schools in NRW, representative of all students in grade 8 at the time of testing in the first half of 2016 (Ziemes et al., 2017).

In this paper, we focus on results regarding the outcome of ‘intended political participation’. With a total of 23 items, students were asked to rate on a four-point Likert scale how likely they would be to participate in certain activities to engage politically as adults, or to express their opinions in the future (ranging from ‘I would certainly do this’ to ‘I would certainly not do this’). Four scales were constructed from these data: intention to participate in elections (e.g. voting in national elections), intention to participate in political organisations (e.g. joining a political party), intention to participate in legal non-formalised activities (e.g. engaging in a peaceful demonstration), and intention to participate in illegal protest activities (e.g. spraying graffiti to express one’s opinion). Internationally, the scores of these scales have been centred to a mean of 50 scale points with a standard deviation of 10 points. Higher scale values indicated a stronger probability of answering the items in a positive way (Schulz, Carstens, et al., 2018).

In order not to overestimate the impact of the local context, two school-related variables from the ICCS data set were chosen as control variables. These were the school type (dichotomised so that 1 = highest, academic track school) and a measure of students’ socioeconomic status (SES). In ICCS 2016, three indices were used to operationalise students’ SES: the highest-ranking occupation of parents, the highest level of education attained by parents, and self-reported number of books at home. Using principal component analysis, one factor was extracted. This national index of socioeconomic background was standardised within each country to a mean of 0 and a standard deviation of 1. Full documentation of the scales and indices can be found in the ICCS 2016 technical report (Schulz, Carstens, et al., 2018). The descriptive statistics of the NRW subsample are reported in Table 1.

**Table 1:** Descriptive statistics of variables in the ICCS 2016 data set (NRW subsample)

Measure	*M*	(*SE*)	*SD*	(*SE*)
Intention to participate in elections	46.5	(0.4)	10.2	(0.2)
Intention to participate in political organisations	47.8	(0.3)	8.5	(0.2)
Intention to participate in legal non-formalised activities	44.9	(0.3)	8.8	(0.3)
Intention to participate in illegal protest activities	46.0	(0.3)	8.7	(0.2)
Index of socioeconomic background	0.0	(0.1)	1.0	(0.0)

*N* = 1451 students 59 schools. Percentage of students in highest school track = 44.5% (*SE* 1.6%). Estimated population = 150,237 (*SE* = 4503). *M* = mean; *SD* = standard deviation; Corresponding standard errors (*SE*) in parentheses

### Data Set 2: Open Data from the Federal State of NRW

In the classification of statistical regions of the European Union, NRW consists of 53 administrative districts at NUTS-3 level (*Nomenclature des Unités territoriales statistiques*, Eurostat, 2020). Additionally, these 53 administrative districts are subdivided into 30 rural districts (*Kreise*) and 22 independent cities (*kreisfreie Städte*) plus the metropolitan region of Aachen, which includes the former independent City of Aachen and a rural district (district of Aachen), but has acted as one administrative district since 2009. These districts represent the lowest level of government and are granted some autonomy at local level for certain public responsibilities, such as school administration, welfare, economic development, and public health. To construct indicators that depict the local context of political socialisation, we drew data from the *Landesdatenbank* (database of the federal state of NRW, IT.NRW, 2020b) as well as the *Kommunale Bildungsdatenbank* (local education database, IT.NRW, 2020a) for each of these administrative districts in NRW. To provide an adequate context for the ICCS 2016 data set, we used data from 2016 or the closest year to 2016. We retrieved a total of 74 data points. These data were related to demographics, employment, income, economic performance, public expenditure, cadastral data, and number of schools, teachers and students. On that basis, 25 indices could be constructed. For documentation of the indices derived from the open data drawn from the administrative and educational databases of the federal state of NRW, see “Appendix 1”.

**Fig. 1:**
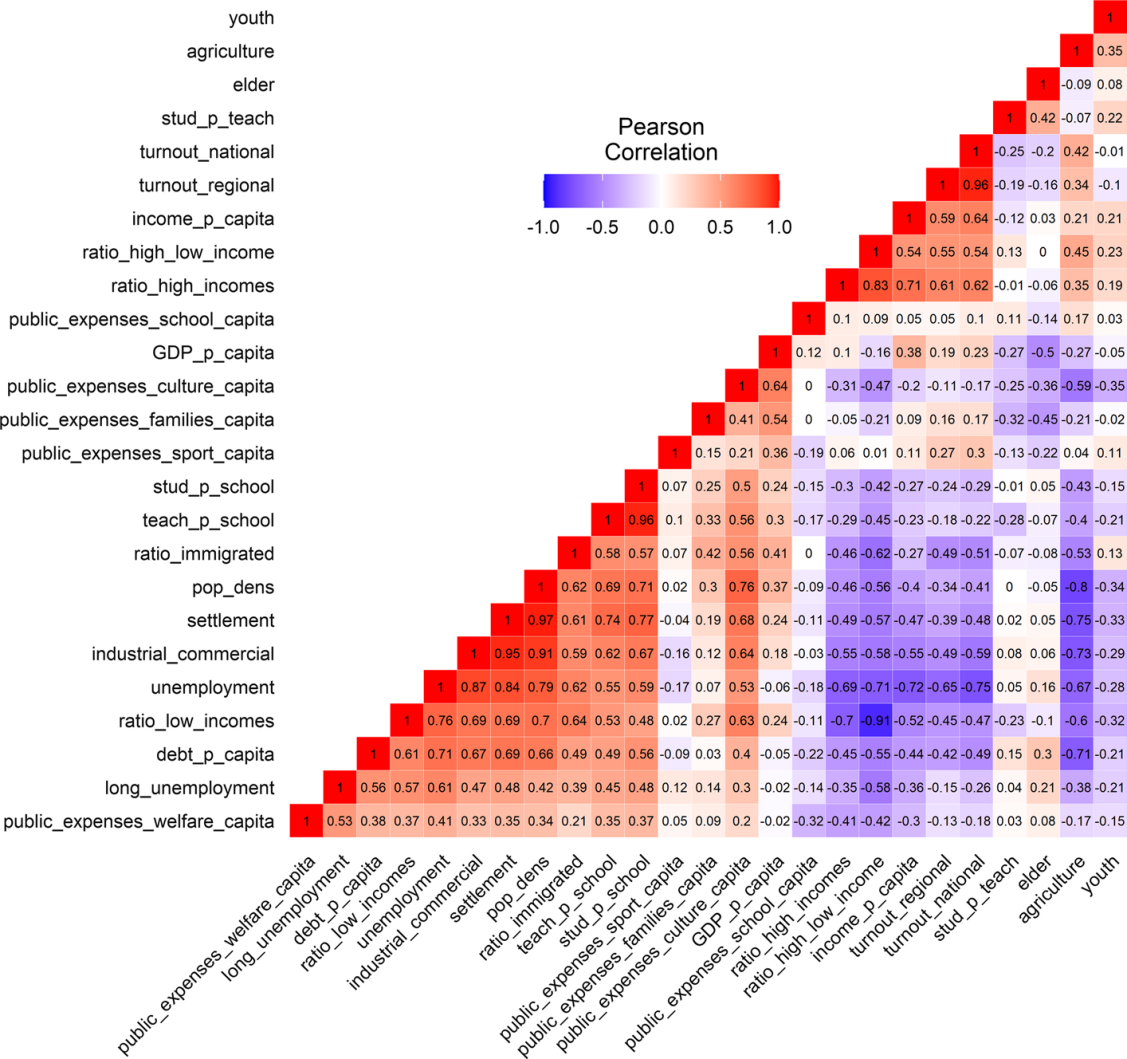
Heatmap of indices derived from open data from NRW

Figure 1 utilises a heatmap to visualise the correlation matrix of these 25 indices. Red fields indicate a positive correlation and blue fields a negative correlation between two values. The more saturated the colour, the stronger the correlation. The numbers represent Pearson’s *r*.

### Procedures

All operations that only affected the open data drawn from NRW databases were conducted in R (R Core Team, 2020). In the first step, we used the open data sources to retrieve indicators to depict the broader local context of political socialisation. The data are stored in multidimensional arrays that can be accessed via the SOAP/XML protocol (DESTATIS, 2020). We used the *wiesbaden* package (Marbach, 2020) to retrieve the data cubes of interest. Using *tidyr* and *dplyr* (Wickham et al., 2019), we converted the retrieved data into a simplified structure that allowed the use of further statistical operations that required one row per unit of information. To address the problem of multicollinearity of these data and to reduce the dimensional structure of the data, we used *psych* (Revelle, 2019) to conduct a principal component analysis (Hair et al., 2014). Map visualisations were conducted with *tmap* (Tennekes, 2018) using open spatial data (Geobasis NRW, 2020).

In the second step, we combined the results of the analyses of the open data with the data set of ICCS 2016. Student data were analysed with multilevel regression models (Raudenbush & Bryk, 2002) to take into account the characteristics of the hierarchically clustered and multistage randomised sample design. These analyses were conducted in *Mplus* (Muthén & Muthén, 2017).

## Results

### Results of the Principal Component Analysis

In order to construct meaningful indicators for depicting the context of adolescents’ political socialisation, we conducted a principal component analysis. This technique allows the examination of underlying patterns in a complex set of interrelated variables. One of the aims of this method is to reduce the dimensional complexity of the data (Hair et al., 2014). We analysed the 25 indices derived from the NRW open data and examined various solutions to determine a feasible number of components to extract from the data. In so doing, we balanced empirical arguments, interpretability, and parsimony (Hair et al., 2014; Revelle, 2020). The three-component solution fulfils these prerequisites. A non-significant chi square of *χ*^2^ = 213.27, *p* > 0.05 and a root mean square of the residuals of *RMSR* = 0.08 indicates that three components are sufficient (Revelle, 2020; Schermelleh-Engel et al., 2003). Table 2 shows the rotated component matrix. The values present the standardised loadings, which can be interpreted as a correlation between an indicator and the respective component. We applied varimax rotation, an orthogonal rotation method to minimise the correlations between the extracted dimensions. To improve readability, we only included loadings greater than *λ*′ >|0.30*|.*

**Table 2:** Principal component analysis of indices derived from open data (*N* = 53 administrative districts)

Name of index	Degree of urbanisation	Aggregated individual wealth	Variety of Opportunity
Settlement	0.81	− 0.43	
Stud_p_school	0.80		
Pop_dens	0.78	− 0.40	0.31
Teach_p_school	0.75		0.32
Debt_p_capita	0.73	− 0.39	
Industrial_commercial	0.70	− 0.56	
Long_unemployment	0.67		
Agriculture	− 0.66	0.34	
Public_expenses_welfare_capita	0.54		
Youth	− 0.37		
Public_expenses_school_capita	− 0.36		
Turnout_regional		0.88	
Turnout_national		0.87	
Ratio_high_incomes		0.79	
Income_p_capita		0.75	
Unemployment	0.64	− 0.72	
Ratio_high_low_income	− 0.41	0.71	
Ratio_low_incomes	0.53	− 0.63	0.33
Ratio_immigrated	0.42	− 0.54	0.41
GDP_p_capita			0.84
Elder			− 0.77
Public_expenses_families_capita			0.70
Public_expenses_culture_capita	0.54		0.65
Stud_p_teach			− 0.56
Public_expenses_sport_capita		0.31	0.33

Standardised loadings based upon correlation matrix. Rotation method: varimax. Only loadings *λ*′ >|0.30|

**Fig. 2:**
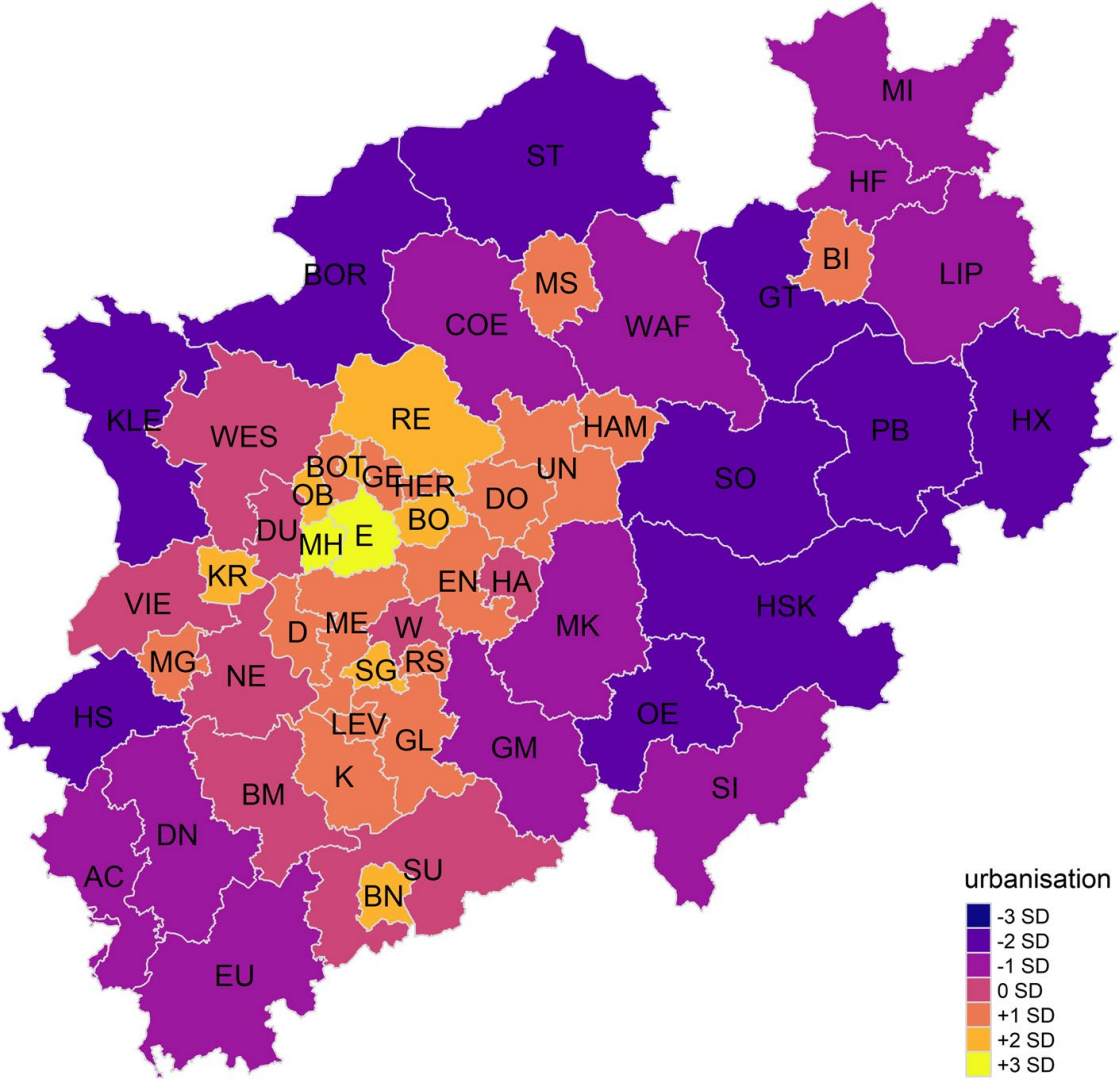
Degree of urbanisation in NRW

The solution provides three clearly distinguishable dimensions of the local contexts of political socialisation. The first component or indicator can be interpreted as *degree of urbanisation.* It is characterised by positive correlations to the ratio of settlement area, population density, school size (more students and teachers per school), and industrial and commercial area. Further, long-term unemployment and higher levels of public spending on welfare are observed. Figure 2 presents the first indicator plotted on a map of NRW. The full names of the administrative districts are provided in “Appendix 2”.

Lighter colours indicate a higher degree of urbanisation compared to the mean of the administrative districts. Each colour represents the value of one standard deviation from the component’s mean; the scales range from − 3 to + 3 SD. While no NUTS-3 region in NRW has a value of -3 SD or lower, the densely populated areas and (former) industrial regions near the rivers Ruhr, Emscher, and Rhine can be clearly distinguished from the more rural regions in the east, north, and south-west of NRW.

**Fig. 3:**
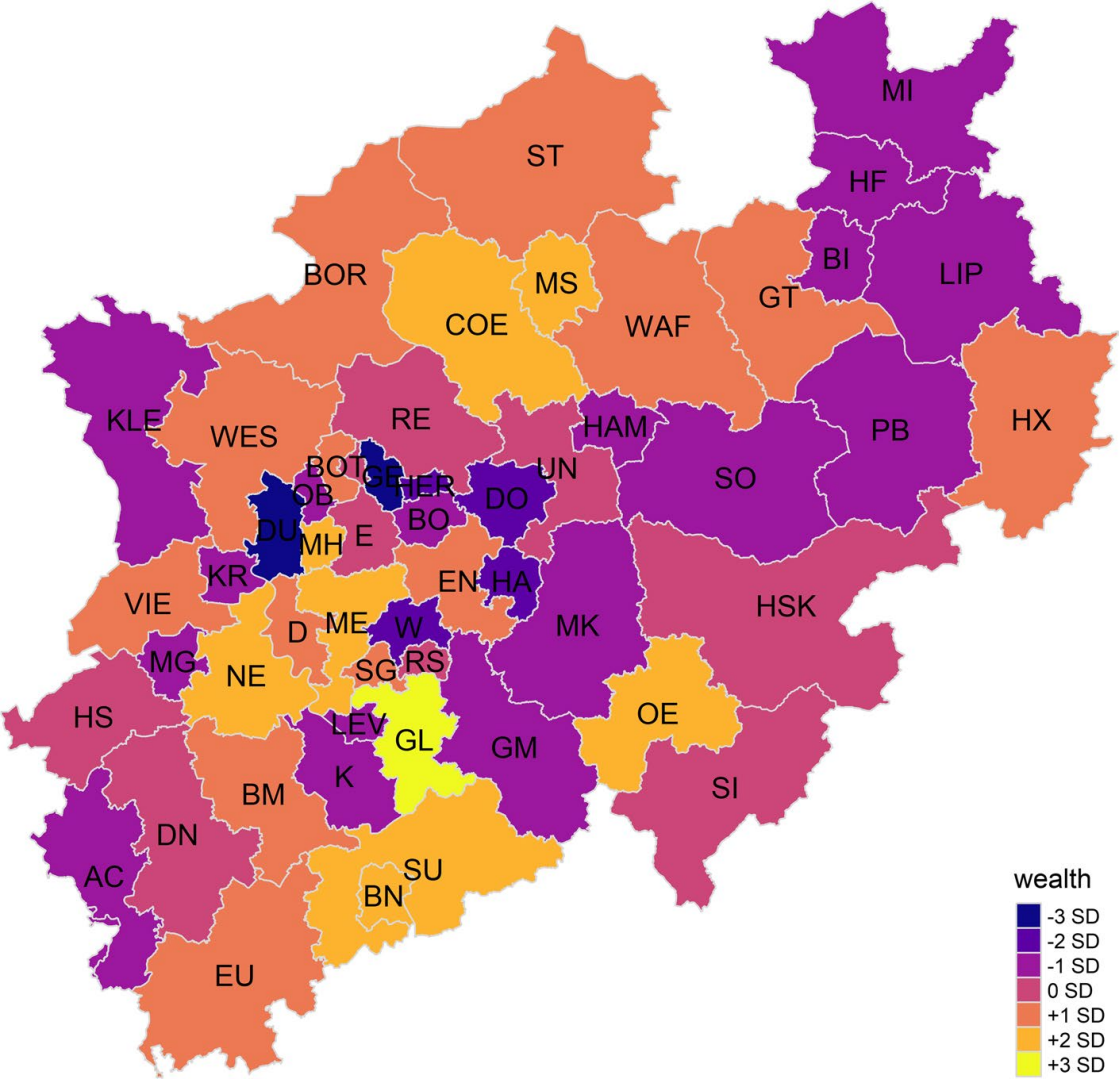
Distribution of aggregated individual wealth in NRW

The second indicator can be interpreted as *degree of aggregated individual wealth*. It is characterised by positive correlations to the ratio of high incomes, ratio of high incomes to low incomes, income per capita, and lower levels of unemployment. Additionally, the indicator is positively related to voter turnout in regional and national elections. The second indicator is plotted on a map in Fig. 3.

The distribution of aggregated individual wealth in NRW does not seem to follow regional patterns. Although the urban core regions of this federal state generally seem to be below average, the cities of Duisburg and Gelsenkirchen stand out with − 3 SD compared to the mean individual wealth in NRW. Notable exceptions, too, are NRW’s capital Düsseldorf, the former federal capital of Bonn, and the city of Münster, which is surrounded by generally richer rural administrative districts in the north of NRW.

**Fig. 4:**
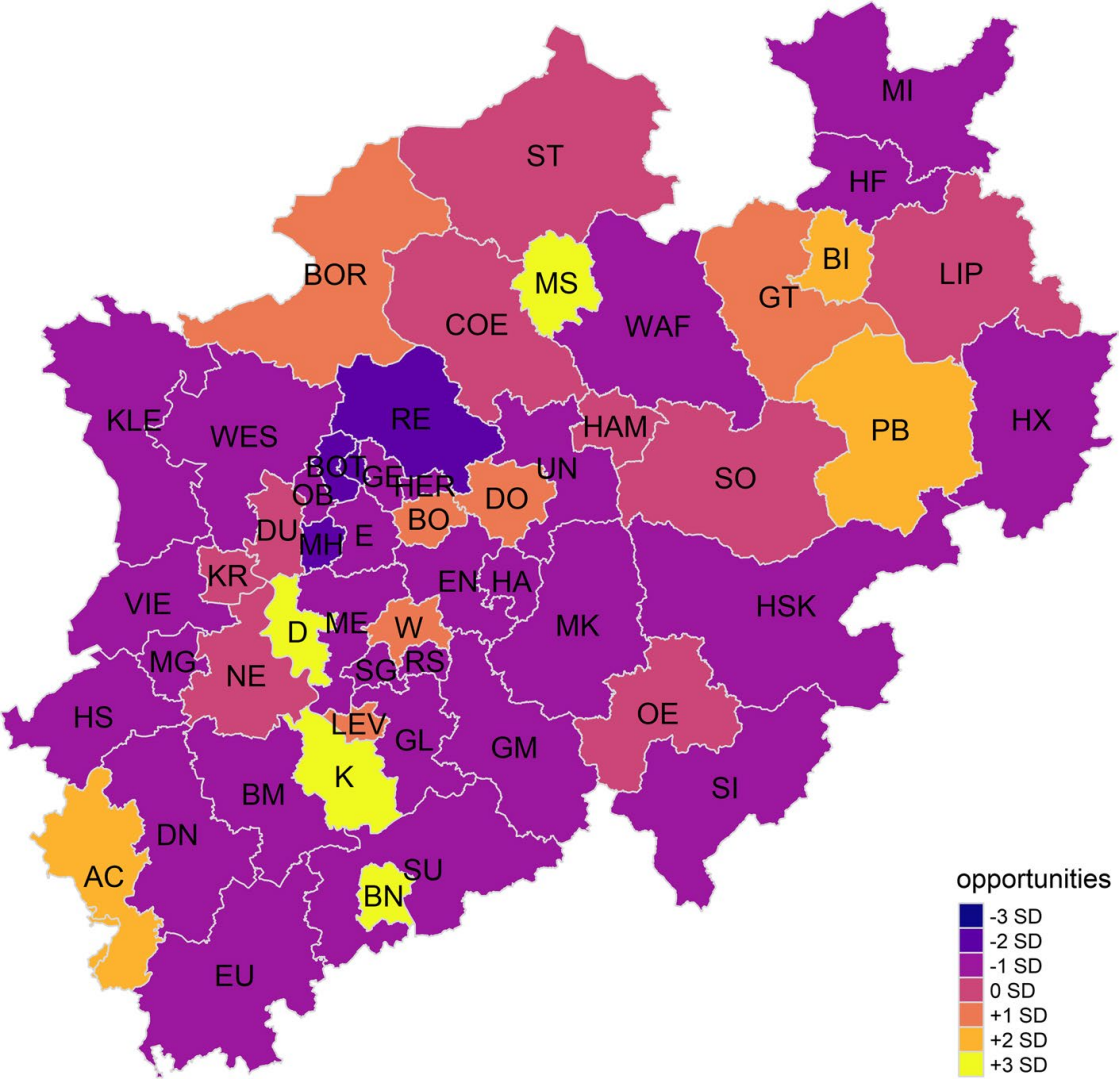
Variety of opportunity in NRW

The third indicator can be interpreted as *variety of opportunity.* It is characterised by its positive correlation to an administrative district’s economic power as indicated by GDP per capita. The higher a unit’s indicator value, the fewer older people live there. This finding is accompanied by higher public expenditure on supporting families, children, adolescents, and culture. The ratio of students to teachers also seems to be more favourable the higher the value of indicator 3. Greater economic power is connected with more and better-paying jobs. More public expenditure in said categories enriches the cultural environment. The third indicator is plotted on a map in Fig. 4.

The cities of Bonn, Cologne, Düsseldorf, and Münster stand out with + 3 SD above the mean variety of opportunity. Large areas of NRW seem to offer subpar opportunities for its citizens, especially the rural Rhine region in the western parts of NRW and the south-eastern hilly areas. However, there does not seem to be a clear pattern of this urban–rural divide: some cities within the former industrial core along the rivers Ruhr and Emscher offer an above-average variety of opportunity (Bochum, Dortmund), while others perform below average (Essen, Mülheim an der Ruhr, Gelsenkirchen, Herne).

In total, the three indicators allow for interesting insights into the sociogeographic structure of a German federal state. Most importantly for socialisation research, the urban–rural divide is not the only source of local differences. Some highly urbanised cities like Cologne with low individual wealth excel in the provision of opportunities for their citizens. Other cities, like Mülheim an der Ruhr, would seem to be more favourable in terms of their citizens’ wealth but fail to translate this wealth into above-average opportunities. Some rural communities can be characterised by high aggregated individual wealth, others by below average wealth. All in all, considering the degree of urbanisation in isolation creates an image of social realities that does not adequately capture the complexity of the sociogeographic contexts in which young people grow up and undergo their process of political socialisation. A three-dimensional approach that describes local environments in terms of their degree of urbanisation, degree of aggregated individual wealth, and variety of opportunity might be more suitable.

### Preliminary Analyses to Prepare Multilevel Model Analysis

The use of multilevel analysis is generally necessary for complex survey data (Hahs-Vaughn et al., 2011). This is because differing amounts of variance of the variables are explained by group membership (e.g. being a student in a certain school). The amount of variance is indicated by the intraclass correlation coefficient (ICC). There are no clear rules regarding the cut-off criteria to justify multilevel analyses; however, an *ICC* > 0.10 can be considered as a medium effect of group membership, meaning that more than 10% of the variance of a variable can be traced back to group level. An *ICC* < 0.01 indicates negligible amounts of variance at group level (Woehr et al., 2015). We therefore decided to conduct analyses only for variables with an *ICC* < 0.10. This includes the measures of *intention to participate in elections* (*ICC* = 0.183) and *intention to participate in illegal protest activities* (*ICC* = 0.101). The measures of *intention to participate in political organisations* (*ICC* = 0.009) and *intention to participate in legal non-formalised activities* (*ICC* = 0.006) were excluded from further analysis as these variables did not have sufficient amounts of variance at group level.

### Results of the Multilevel Model Analysis

To analyse the relationship between the local context of political socialisation and adolescents’ intention to participate politically in the future, we evaluated two multilevel regression models for the dependent variables: one for *intention to participate in elections* and one for *intention to participate in illegal protest activities*. The indicators of *degree of urbanisation*, *degree of aggregated individual wealth*, and *variety of opportunity* were introduced as independent variables. Further, we included two control variables in the analyses to account for the effects of NRW’s tracked school system: the mean SES of each school class, and the school type. Table 3 reports the results of these analyses. For each of the two independent variables, we report the standardised regression weights and the respective statistical significance. Furthermore, Table 3 shows the variance at the between level (*R*^2^), the *ICC*, and the average cluster size.

**Table 3:** Results of multilevel analysis (between level only, *N* = 53 schools)

	Intention to participate in elections			Intention to participate in illegal protest activities		
	*β*	*SE*	*p*	*β*	*SE*	*p*
*Indicators*						
Individual wealth	0.17	(0.10)	0.07	− 0.08	(0.13)	0.56
Variety of opportunity	− 0.34	(0.13)	0.01	0.43	(0.17)	0.01
Degree of urbanisation	− 0.15	(0.10)	0.12	0.13	(0.09)	0.14
*Control variables*						
SES (class mean)	0.38	(0.16)	0.02	− 0.34	(0.22)	0.13
School type (1 = highest track)	0.42	(0.19)	0.03	− 0.33	(0.20)	0.11
*R*^2^ (between)		0.67			0.56	
ICC		0.18			0.10	
Average cluster size		23.6			23.6	

*β* Standardised regression coefficient, *SE* Standard error, *p* Probability of type I error (two-tailed), *R*^2^ Determination coefficient, *ICC* Intraclass correlation coefficient of dependent variable

Only one of the three indicators used to describe the local context of political socialisation was significantly correlated with *intention to participate in elections*, and that was *variety of opportunity*. There is a moderate negative effect of variety of opportunity (*β* =  − 0.34, *p* < 0.01): the higher the economic power of a NUTS-3 region, and the relatively more public expenditure is allocated to supporting families, adolescents, and cultural activities, the less likely it is that students in grade 8 intend to participate in elections as adults. *Degree of urbanisation* did not seem to affect the measure of intended participation in elections significantly (*β* =  − 0.15, *p* > 0.05). *Aggregated individual wealth* in an administrative district does, however, seem to be a borderline case (*β* = 0.17, *p* < 0.10); a follow-up study with more level-2 units would be desirable to investigate if the insignificance of this indicator is a robust effect or whether it is due to the relatively small number of school classes in our sample. When controlling for the stratification effects (average SES at class level, *β* = 0.38, *p* < 0.05 and school type, *β* = 0.42, *p* < 0.05), the full model explains 67% of the level-2 variance of the independent variable, which can be interpreted as a large effect (Cohen, 1988).

The results of the analysis of the measure of *intended participation in illegal protest activities* complement the above results. *Variety of opportunity* in an administrative district is moderately positively correlated with the independent variable (*β* = 0.43, *p* < 0.05). Neither *degree of individual wealth* (*β* =  − 0.08, *p* > 0.05) nor *degree of urbanisation* (*β* = 0.13, *p* > 0.05) have a significant impact on adolescents’ intentions to participate in illegal protest activities. Also, the control variables SES (class mean) and educational track had no significant correlation with intended illegal protest activities. The full model explains 56% of the level-2 variance of the independent variable, which can again be interpreted as a large effect.

## Discussion

The principal component analysis supported a three-dimensional approach which allowed a description of the NUTS-3 regions in terms of their degree of urbanisation, degree of aggregated individual wealth, and variety of opportunity. This differentiation seems to be especially important for describing differences in the contexts of political socialisation. *Degree of urbanisation* was not suitable for explaining any differences in the intended political behaviours of students in grade 8 in NRW. *Degree of aggregated individual wealth* was not suitable either, although a re-examination of this potential correlation might lead to promising results if a larger data set was used in a possible follow-up study of ICCS. The only relevant characteristic of the local context of political socialisation was *variety of opportunity*, showing an interesting mirroring pattern. The higher the measure, the less frequently students intend to participate in elections as adults. At the same time, the higher the measure, the more often students intend to participate in illegal protest activities.

Administrative districts with a higher variety of opportunity have more to offer to young people: a high GDP per capita might indicate more and better-paying job prospects. At the same time, the higher GDP might be correlated with higher tax revenue, which allows for more public expenditure on supporting families, children, and adolescents, as well as cultural activities. Concomitantly, the lower relative proportion of older people means that there might be a higher demand for public expenditure in these fields in the given districts. These environments might be more attractive for younger people, have a richer cultural life, and offer more non-traditional lifestyles. Interaction in these environments that have a higher variety of opportunity might *mobilise* (Verba & Nie, 1972) for certain alternative means of political participation, closely related to the mechanism Milbrath (1972) described. If such alternative means of political participation are more commonly observed in these environments, they may act as stimuli that encourage individuals to participate in similar ways. A greater number of younger people with more non-traditional lifestyles might also be correlated with a higher likeliness of encountering social support for non-traditional means of political participation; social norms differ. For instance, graffiti as an expression of political opinions emerges in certain urban environments and is accompanied by a subculture that revolves around graffiti (e.g. Kindynis, 2018). Further research could also try to assess if new social movements like ‘Fridays for Future’ show more intense activity in environments with a higher variety of opportunity. Of course, the emergence of environment advocacy groups could also be related to a higher prevalence of responsible environmental practices in certain spaces. Deimel and Buhl (2017) observed differences at country level in students’ perception of problems related to pollution and climate change. They hypothesised that these differences might be explained by regional differences in the visibility of specific problems. However, data availability limits the testing of this hypothesis, especially when examining smaller regional entities such as NUTS-3 regions.

In contrast to these results, more conventional political participation seems to emerge in environments with a lower variety of opportunity, where alternative means of participation might feel less ‘normal’ and therefore receive less social support. Not all urban environments offer a high variety of opportunity, which is also the reason that the size of the urban environment was no reliable variable for explaining the differences in outcomes of political socialisation in the past (Schulz et al., 2018b). A better differentiation of the local context of political socialisation as exemplified by our research could help to improve the theoretical frameworks of large-scale assessments like ICCS. Further, our analyses reveal that combining data from different public, open, and/or official sources is a viable approach for addressing the lack of broader contextual data in educational research, provided that enough data are available to observe significant statistical relationships.

There are some limitations to our study. First, data availability was a limiting factor for our results: while 59 clusters would seem sufficient, a higher number of clusters would enable a more precise estimation of the between cluster variation in the outcome variables (Austin & Leckie, 2018). Second, we only examined the case of NRW. While this state offers a wide spectrum of large urban and rural communities that differ in their sociostructural and sociocultural features, our results cannot be reliably generalised for other regions. A comparison of different regions would be desirable but is made difficult by the lack of available comparable data. A third factor which may potentially limit the replication of our present study is the impact of the COVID-19 pandemic. Local policy makers will face considerable challenges in addressing educational inequalities that may have been exacerbated by lockdowns and prolonged remote learning from home with potentially very unequal access to resources and support (van Lancker & Parolin, 2020). As of now, it is impossible to assess in which way such impacts might affect processes of political socialisation in the future, especially regarding the observed effects of the variety of opportunity on the NUTS-3 level.

In conclusion, the question arises to what extent educational policy making has to take into account the results presented here when aiming to improve the quality of civic education. Educational policy in Germany is mainly set at federal state level, in this case NRW. However, our analyses show that such high-level policy making does not prevent entities at NUTS-3 level from developing meaningful differences in contextual factors that, in turn, affect political socialisation. With that, it must be discussed if educational policy making can address these contextual differences at all and, if so, how and by whom this should be done. Clearly the differences in structured opportunities that we show to be effective for the political socialisation of adolescents are only partly decided at NUTS-3 level. For instance, whether a university or a cultural institution of national importance is located in a specific town is not decided at local level; however, local authorities *can* determine and promote how children and young people get access to cultural institutions like theatres, museums, and heritage sites. This duality again strongly calls for targeted assessments of contextual information when interpreting the results of large-scale assessments of education policy and outcomes in future study cycles.

## Data Availability

The data availability is documented in a separate data deposition information document.

## Appendix 1: Description of Indices Derived from Open Data from Governmental Entities in NRW

**Table:**

Index name	Description	Data
Pop_dens	Ratio of people to total area	Total population derived from amount of people in all age groups (17 data points) and cadastral data (4 data points)
Ratio_immigrated	Ratio of people with immigration background to total population	Population data (18 data points)
Youth	Ratio of young people (14 and younger) to people of employment age	Amount of people in various age groups (15 data points)
Elder	Ratio of elder people (65 and older) to people of employable age	Amount of people in various age groups (13 data points)
Agriculture	Ratio of area used for agricultural purposes to total area	Cadastral data (5 data points)
Settlement	Ratio of settlement area to total area	Cadastral data (5 data points)
Industrial_commercial	Ratio of area used for industrial or commercial purposes to total area	Cadastral data (5 data points)
Stud_p_teach	Ratio of students to teachers	Amount of students and teachers (2 data points)
Stud_p_school	Ratio of students to schools	Amount of students and schools (2 data points)
Teach_p_school	Ratio of teachers to schools	Amount of teachers and schools (2 data points)
Turnout_national	Voter turnout in national elections 2017 (Bundestag)	Amount of voters and eligible voters (2 data points)
Turnout_national	Voter turnout in regional elections 2017 (Landtag)	Amount of voters and eligible voters (2 data points)
GDP_p_capita	GDP per capita	(1 data point)
Debt_p_capita	Public debt per capita	(1 data point)
Income_p_capita	Net income per capita	(1 data point)
Unemployment	Ratio of unemployed people	(1 data point)
Long_unemployment	Ratio of people who are unemployed for one year or longer to total unemployed people	Amount of unemployed people (2 data points)
Ratio_high_low_income	Ratio of households with high net income to households with low net income	Amount of households by net income class (4 data points)
Ratio_high_incomes	Ratio of households with high net income to total households	Amount of households by net income class (10 data points)
Ratio_low_incomes	Ratio of households with low net income to total households	Amount of households by net income class (10 data points)
Public_expenses_school_capit	Public expenditure on schooling per capita	Sum of expenditure items related to schooling (11 data points), population data (18 data points)
Public_expenses_culture_capita	Public expenditure on culture per capita	Sum of expenditure items related to culture (9 data points), population data (18 data points)
Public_expenses_families_capita	Public expenses for support of families, children and adolescents per capita	Sum of expenditure items related to supporting families, children, and adolescents (6 data points), population data (18 data points)
Public_expenses_sport_capita	Public expenditure on support of sports per capita	Sum of expenditure items related to supporting sports (2 data points), population data (18 data points)
Public_expenses_welfare_capita	Public expenditure on basic needs of welfare recipients per capita	Sum of expenditure items related to supporting the basic needs of welfare recipients (1 data point), population data (18 data points)

## Appendix 2: List of Administrative Districts in North Rhine-Westphalia

**Table:**

Abbreviation	Name	Type (1 = rural district; 2 = independent city)
AC	Städteregion Aachen	1,2
BI	Bielefeld	1
BM	Erftkreis	2
BN	Bonn	1
BO	Bochum	1
BOT	Bottrop	1
BOR	Kreis Borken	2
COE	Kreis Coesfeld	2
D	Düsseldorf	1
DN	Düren	2
DO	Dortmund	1
DU	Duisburg	1
E	Essen	1
EN	Ennepe-Ruhr-Kreis	2
EU	Euskirchen	2
GE	Gelsenkirchen	1
GL	Rheinisch-Bergischer-Kreis	2
GM	Oberbergischer Kreis	2
GT	Gütersloh	2
HA	Hagen	1
HAM	Hamm	1
HER	Herne	1
HF	Herford	2
HS	Heinsberg	2
HSK	Hochsauerlandkreis	2
HX	Höxter	2
K	Köln	1
KLE	Kleve	2
KR	Krefeld	1
LEV	Leverkusen	1
LIP	Kreis Lippe	2
ME	Kreis Mettmann	2
MG	Mönchengladbach	1
MH	Mülheim an der Ruhr	1
MI	Minden-Lübbecke	2
MK	Märkischer Kreis	2
MS	Münster	1
NE	Rhein-Kreis Neuss	2
OB	Oberhausen	1
OE	Olpe	2
PB	Paderborn	2
RE	Recklinghausen	2
RS	Remscheid	1
SG	Solingen	1
SI	Siegen	2
SO	Soest	2
ST	Steinfurt	2
SU	Rhein-Sieg-Kreis	2
UN	Unna	2
VIE	Viersen	2
W	Wuppertal	1
WAF	Warendorf	2
WES	Wesel	2
